# Conducting transformative research protocols in sports science

**DOI:** 10.3389/fspor.2025.1646717

**Published:** 2025-10-07

**Authors:** Robin Santi, Simon Isserte, Cyrille Gaudin, Sébastien Chaliès

**Affiliations:** ^1^ Laboratoire Interdisciplinaire de Recherche en Didactique, Éducation et Formation (LIRDEF), Faculty of Education, University of Montpellier, Montpellier, France; ^2^ Éducation et Diversités en Espaces Francophones (FrED), National Higher Institute for Teaching and Education, University of Limoges, Limoges, France

**Keywords:** researcher-practitioner partnership, transformative research, methodology, sports science, transdiciplinary

## Abstract

This article presents a methodology for “fundamental field research” in sports science, from a transdisciplinary and transformative perspective. It is based on a close and symmetrical partnership between researchers and practitioners. The approach is structured around four detailed and illustrated methodological stages: (1) researchers develop an auxiliary hypothesis derived from a stabilized theoretical framework; (2) practitioners and researchers co-design an experimental training set-up that puts this hypothesis to the test; (3) this set-up, designed to disrupt ordinary professional practices, is implemented and analyzed; (4) the set-up's spin-offs are identified, both scientifically for the researchers and p*rofessionally* for the practitioners. This methodology creates a consubstantial relationship between scientific and professional aims, encourages co-production of knowledge, and questions the relationship with sustainability in a field where the demand for immediate performance tends to curb experimental dynamics.

## 1 Introduction: the researcher-practitioner partnership in sports science

Building and implementing a partnership between researchers and practitioners means first and foremost looking at the delicate convergence of their respective concerns. The scientific aims (e.g., to produce knowledge) prioritized by researchers sometimes clash with the technological aims (e.g., to develop practical solutions to professional problems) valued by practitioners. This difficulty is exacerbated by the fact that the demands of high-level sport usually leave little room for experimentation, given the urgency of results and the stakes of competition: as Rynne and Mallett [(1), p. 23] write, although this is qualified later, “the “win at all costs” ethos that permeates much high performance sport seems at direct odds with the notion of sustainability.” The consequences are numerous. Fernández-Fernández and Santos Rosa (2) point out, for example, that sports training is often still based on old beliefs and anecdotal evidence provided by the practitioners themselves, with no scientific basis. In the same vein, Fullagar et al. (3) point out the recurring difficulties linked to the funding of this type of research, the delicate adhesion of practitioners to it and their partial understanding of the research questions involved.

To overcome these difficulties, new forms of research are emerging that promote partnerships between researchers and practitioners. At one pole, *evidence-based practice* (4) pushes research towards *applied sport science* relying on *embedded scientists* who, integrated into sport staffs, mediate between theory and practice so as to reduce the weight of beliefs in decision-making (5, 6). At the opposite pole, a second paradigm calls for *bridging the gap* between the field and science values *insider knowledge*, i.e., the situated knowledge of practitioners, still too often neglected by academic research (7). The aim is to achieve greater objectification of the problems encountered by practitioners, so that scientists can more easily take them up as research objects. Between these two orientations, *collaborative action research* seeks to explicitly merge the perspectives of both worlds by involving practitioners as co-researchers. Widely used in rugby [e.g. (8)], this type of partnership contributes to greater integration of the scientific results produced into practice.

Following on from this work, we propose here to detail another paradigm for underpinning the partnership between researchers and practitioners in a different way: transformative research, conducted *with and for* practitioners. After a brief description of this scientific approach from an epistemological point of view, the methodological conditions to be met are set out and illustrated in four stages. They open the discussion on the issues underlying the conduct of such research protocols in sports science.

## 2 A new approach to sport science research

Epistemologically speaking, the transformative research described here is rooted in a process of (in)validation of auxiliary hypotheses based on a stabilized theoretical framework that can be likened to a stable theoretical “hard core” (9). The scientific approach therefore consists, initially, of scientists constructing an auxiliary hypothesis based on which they can then go out and meet practitioners. In this meeting, the scientists work with the practitioners to see how they can temporarily “transform” their day-to-day practice, in the form of a field experiment, so as to be able to test the validity of their auxiliary hypothesis. At the same time, they try to take account of the practitioner's request(s) for help. In the context of this negotiation work, which results in an “interfacial” object of study (10), the scientific dynamic remains “consubstantial” with the professional dynamic (11). Although they respond to intrinsically distinct logics—scientific production, on the one hand, technological innovation on the other—these dynamics are inseparable and underpin the transformative nature of “fundamental field research” (12).

For any transformation to be heuristic for both scientists and practitioners, certain methodological conditions must be met. These can be broken down into four successive stages. These stages are described in detail below and illustrated on the basis of work carried out as part of a “research program” in cultural anthropology (13). This framework is grounded in Wittgenstein's philosophy of action and assumes that human conduct is mediated by language or, more specifically, by the culturally embedded of rule-following (14).

### 2.1 Stage 1: A scientific transformation to initiate the experimental training program

The examples given in this stage are taken from Robin Santi's doctoral thesis (in progress),^1^ which is being conducted in youth rugby. As part of this research work, the interest of which for practitioners was to implement ethics in their ordinary coaching practices with young people, a review of the scientific literature first made it possible to identify the links between the educational virtues of youth sport, their compatibility with competitive practice, and the role played by coaches in this dynamic [e.g. (15, 16)]. This phase of the work enables the researcher to define his object of study with the necessary scientific rigor. Following on from this, on the theoretical level, the primary need was to give “ethics” a foothold, this time as an object of training, by bringing it closer to the “rough ground of ordinary life” (17). Rather than being approached as a set of abstract principles, it was seen in its pragmatic dimension, i.e., in direct contact with practices in the field. For the specific purposes of this research, the challenge lay in hybridizing the culturalist framework with the philosophy of John Dewey (18), and his concept of the “community in miniature” (p. 41). This movement made it possible, in this context, to trace the contours of “ethics” as a form of democratic life, valuing problem-solving, cooperation and a sense of community. Through a theoretical stabilization of *what ethics is*, it became possible to implement it in practice. Rugby, a team sport based on the values of utility and camaraderie, offered a particularly favorable context for this type of research.

As emphasized above, the starting point for all transformative research remains its fundamentally scientific nature, embodied in the testing of an auxiliary hypothesis. In this doctoral work, Robin Santi theoretically constructed the hypothesis that by conducting the experimental long-term training program with the practitioners, getting them to co-construct with the researcher rugby learning rules charged with a sense of community, and by creating a situational background giving meaning to these rules, their professional development would be enhanced. Formalizing the auxiliary hypothesis naturally leads to the development of the experimental training program and contractualization with the practitioners.

### 2.2 Stage 2: co-constructing the transformation to test the pre-defined scientific hypothesis

It is then necessary to delimit the professional field in which, through negotiation with the practitioners, the researchers can test the heuristic nature of their auxiliary hypothesis. This involves defining an “interface” object of study, at the crossroads of scientific and technological aims. To achieve this, several meetings are organized to enable researchers and practitioners to co-construct a transformation of the practitioners' usual practices. Indeed, while researchers work from a “theoretical framework”, professionals also have their own specific conceptual framework (called here “professional framework”). This framework comprises all their preoccupations, knowledge, skills and beliefs. We will illustrate this stage using a research protocol^2^ set up in partnership with a football coaching training organization, and which has been the subject of several publications [e.g. (20)]. The auxiliary hypothesis supporting this study was as follows: digital technologies, if used appropriately, could make it easier to consider the complexity of the training object “collective action”. This hypothesis was defined in terms of the difficulties encountered by coaches in dealing with the inherent complexity of collective actions when it comes to building them in players.

The twofold challenge was to characterize a collective action through its theoretical conceptualization, combined with technological tools that would enable suitable training activities to be implemented. To do this, the researchers set out to modify the practices of the coaches by proposing unique and articulated uses of different digital technologies (e.g., teaching a collective action using a 3D video animation, supporting the learning of this action using time-lag video). The researchers took care to test the various activities proposed, particularly from a technological point of view, to ensure that they were compatible with the material and financial resources of the structures in which these coaches operate. In the end, it was because they saw an opportunity to learn about innovative uses of digital technologies, within “acceptable” constraints in terms of their involvement, that the coaches agreed to take part in this experimental training program.

The various negotiation meetings between the researchers and the practitioners also provided an opportunity to specify certain conditions relating to the involvement of each party in the program. Among these conditions, the time cost involved in taking part appeared to be particularly significant. This is specific to the field of sport, as the amateur context does not always allow the necessary conditions to be met (e.g., uncertain material conditions, organizational difficulties, level of involvement of sportspeople in their project) for the experiment to be carried out in full. Conversely, top-level sport offers conditions that are *a priori* more favorable to the implementation of transformative research protocols (e.g., conditions of practice, level of supervision). However, the stakes inherent in top-level competition often act as a brake on commitment to this type of transformative approach, shaking up the habits of practitioners in a world where stability is essential for day-to-day performance. In the end, it is undoubtedly an intermediate level of practice that seems most conducive to implementing this type of protocol.

### 2.3 Stage 3: supporting the transformation during the implementation of the experimental training program

During this stage, a strong methodological singularity is asserted: it is the movement of transformation of the practitioners' activity that becomes the object of analysis. For the researcher, therefore, it is not a question of observing a stabilized practice, but of grasping a process that is situated, moving, and profoundly linked to the specific modalities of the experimental set-up. In this sense, the transformation underway cannot be reproduced as it stands; it provides a heuristic opportunity to analyze the production of knowledge *in situ*.

Thus, during the actual transformation of the practitioners' ordinary activity, the collection is done in two stages and yields two types of data. First, the audiovisual recording of training and/or competition situations makes it possible to collect so-called “extrinsic” data, which correspond to the data reflecting the activities carried out *in situ*. On this basis, interviews confronting the practitioners with the traces of their situated activities are carried out and recorded. They provide what we call “intrinsic” data, which correspond to the verbalizations produced *post actu* on the teleology of the activities observed. These interviews, known as self-confrontation interviews (21), have a dual purpose: to identify and formalize the practitioners' practical reasoning after the event (22), by “allowing themselves be informed by them” (36) as to the meaning they associate with their past activities, and also to follow up the transformation of the practitioners initiated in the previous stage. During this interview, the practitioners not only give meaning to their past activities but also transform themselves by addressing them to the researcher. Borrowed from the work of Santi and Chaliès (23), the example detailed below shows the interaction between the researcher and the *post-actu* practitioner, but above all the transformative dimension at play during this interview. The youth coach's verbalization during the self-confrontation interview enables him to inform the researcher about the reality of his activity of accompanying the training situation, and himself to enter into a reflective—and transformative—process about this activity (Table 1). In this case, the coach gradually shifts from a justificatory framework grounded in the rule's mere application to one oriented toward meaning-making. Such a transformation is emblematic of a professional development process.

**Table 1:** Illustration of the transformation of the youth coach's practical reasoning.

Start of the program—Training session 2	End of the program—Training session 6
Extract from a self-confrontation interview	Extract from a self-confrontation interview
Researcher (R): At that moment in your coaching, you see this kick… you weren't expecting it. And, well, it's definitely not the brightest idea… and beyond the idea, it's poorly executed on top of that. So how do you handle it? Coach (C): In my role, it's about… working with it. What I really want is for them to play, put some speed into it at the very least, but not make that kind of choice. This time he gets away with it because he throws in a sidestep, then cuts back inside, and the four defenders are all beaten… but I’d really prefer him to play it differently. The problem is, I should stop, but I can't. So I guide him as he goes so that he reflects on it in the moment. Was that choice really the right one? Because the opportunity wasn't really there. If he really thinks about the situation… the kid being smart—that's what we’re looking for—given what was in front of him… Yeah, I think he got it. R: And the fact that you didn't stop the play, but let it keep going to the end—why? Because last week you did stop it. C: Right. That used to be my way of doing things: “you have to explain it and put words on the action.” Uh… But here, since it actually worked out… In the end what we want is for the ball to cross the line. The technique, how it got there—whether it was messy, whether the ball bounced four times or whatever—well, what we want is for it to result in a try, that's the idea.	R: There, the ball comes back through you? C: Yeah. Well, I get in the way, I’m running the drill… but here it's really just total chaos, so I don't want to stop the play. Because he's right to… well, I’m in the middle, but that's just how it's supposed to be played! Except, well, that's no big deal that it doesn't come off, but the intention is good. And so I don't want to punish it, blow the whistle, say “come on,” or “why are you giving me the ball”… That's too punitive, because the idea—the choice—was actually good. […] Sometimes it's a bit messy… But really what we’re after is… If the execution isn't there, it doesn't matter. At ten or eleven years old, you can have mistakes, knock-ons, whatever… What we want is to create momentum. So if you blow the whistle every time some kid's shoelace is untied, that's all you end up doing—they learn nothing, there's no support.
Practical reasoning	Practical reasoning
In situations where a player kicks even though “the opportunity wasn't really there,” but where ‘he gets away with it,’ the idea of valuing the player's initiative concretely means ‘working with it,’ ‘guiding him as he goes.’/The expected outcome is to make him ‘think about the situation’ and to call upon ‘the kid being smart,’/because ‘that's what we’re looking for.’/This also makes it possible to ‘not stop the play’/because ‘I can't’/even though ‘that used to be my way of doing things,’/and because ‘it actually worked out.’/Ultimately, the point is ‘for the ball to cross the line, for the situation to result in a try.’	In situations where ‘it's really just total chaos” and where ‘I get in the way,’ the idea of ‘running the drill’ concretely means making the ball circulate./The expected outcome is to ‘not stop the play’ and to ‘create momentum’/because ‘the intention is good,’ that ‘at ten or eleven years old, you can have mistakes,’ and that ‘if you blow the whistle every time some kid's shoelace is untied, that's all you end up doing—they learn nothing,’ and ‘that's too punitive.’

The processing of the data collected is organized to capture the transformation of the practitioners during the course of the experimental program implemented. To do this, the various practical reasonings are formalized, and their evolution over the course of the experiment provides information about its impact on the practitioners' actual practices and the meanings they associate with them.

Although practitioners need to be acculturated to the “game” of the self-confrontation interview in order to collect relevant data, it is understandable that this type of interview is regularly used as a tool for optimizing training practices, and therefore for purposes unrelated to research (24, 25).

### 2.4 Stage 4: scientific and technological progress

This fourth stage marks the singularity of the processes of constructing new knowledge in which researchers and practitioners are involved. While engaging in such a research process offers them opportunities for shared reflectivity, the benefits they derive from it are specific to them, given their respective concerns. The results produced by the experimental training program make it possible for the researchers to envisage the production of knowledge, while at the same time the practitioners find solutions to the initial questions that led them to become involved in this research protocol.

On the researchers' side, empirical results generate new knowledge and allow the validation (or not) of the auxiliary hypothesis initially adopted. For example, the hypothesis at the origin of Isserte's thesis work (2022), mentioned above, was validated in the light of the results produced. In concrete terms, the training offered to the coaches, which was based on a conceptualization of training resulting from this support research program (13), enhanced their ordinary coaching activities and ultimately the learning of collective actions by their players.

For the practitioners, the transformation was less scientific than professional. In this study, the practitioners took hold of the experimental program that supported the scientific study and, given their own involvement as participants in the research, planned to disseminate it to the wider professional community. Underpinned by the scientific results produced, this so-called “technological” progression (11) provides a new opportunity for transformation for the practitioners who, faced with the need to construct the conditions for deploying the experimental device in an ordinary training context, have engaged in an in-depth reflective practice.

Over and above the use of digital technologies that some coaches were able to re-exploit by adapting them to their context (e.g., depending on the level of practice, the structuring of their training plans), it seems worth emphasizing here the originality of such a research protocol in terms of technological progression at several levels. For example, this study has highlighted the fact that disseminating the experimental program to ordinary training contexts requires significant adjustments in terms of coach training. This is illustrated by the methods used to support coaches in training (e.g., specific support for the tutor via situations simulating the use of these technologies directly on the course site), which are more conducive to transforming the ordinary activities of coach trainers. And that's how the practical implications of this type of research can be considered.

**Figure 1:**
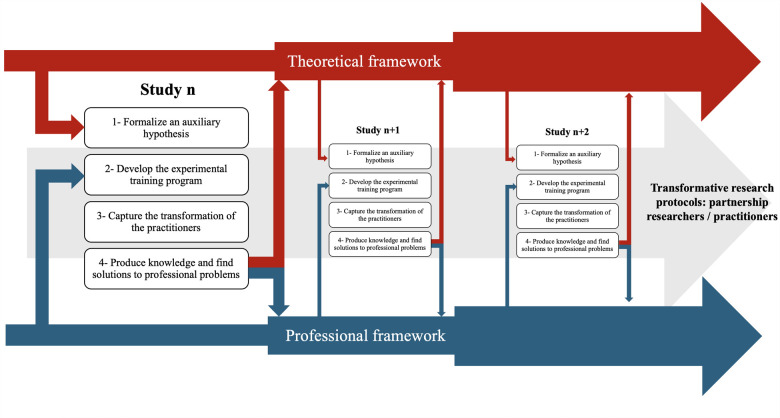
Overview of the transformative research methodology proposed.

Figure 1 provides an overview of the methodological implications of these four successive stages and emphasizes the iterative nature of this transformative research protocol. In more fundamental terms, the empirical results of the study allow the transformation of the theoretical hard core through the validation (or not) of the initial hypothesis. This intrinsic dynamic of the theoretical hard core (9) leads to the formalization of a new hypothesis that should give rise to a new study.

## 3 Discussion

The four methodological stages detailed and illustrated above highlight the way in which researchers and practitioners work together to construct new knowledge within the framework of a transformative research protocol as advocated here. Our proposal is therefore in line with research that has already recognized and understood the complexity of sport-related phenomena by mobilizing knowledge from a variety of disciplines [e.g. (24–26)]. Although this work opens original perspectives to enrich, in particular, the stage of data collection and processing that we are proposing, they remain, for the most part, in an *interdisciplinary* register, in the sense that they bring together researchers from different backgrounds, without necessarily involving practitioners in the formulation of auxiliary hypotheses.

As such, this proposal calls for a more participatory approach, one that does not simply consist of crossing methods or disciplinary perspectives, but aims to co-produce knowledge with practitioners, based on issues arising from their situated experience, and through a transformative movement that “disrupts” their ordinary practices. Such an approach is particularly heuristic when it comes to understanding *wicked problems*, which are characterized by their dependence on context, their evolving nature and the fact that they do not invite a single solution. This is particularly the case in sports science, for example when the aim is to develop players' moral sense or their creativity (27). In these situations, the production of knowledge cannot be confined to a simple juxtaposition of disciplinary perspectives. Following on from work in other fields [e.g. (28)], it is therefore possible to describe this type of research as *transdisciplinary*. Transdisciplinarity certainly means “moving beyond disciplinary silos” (29), but also “situat[ing] the inquiry at the core of the research program, not the discipline” [(30), p. 5]. In practical terms, this means linking problems in the field with academic questions in order to contribute simultaneously to societal and scientific progress. From this perspective, in which the ways in which knowledge is produced are examined in the light of practical issues and the expectations of practitioners, the partnership calls for scientists and practitioners alike to adopt a critical and reflective stance. This dynamic illustrates the tension highlighted by Jahn et al. (31) between the quest for scientific truth and the imperative of utility.

Beyond the methodological aspects, however, what is at stake is a more profound transformation of how knowledge is produced and appropriated. This transformation questions not only the researcher's posture, but also the capacity of the sports field to open up to experimental and uncertain dynamics. While the option of resorting to the *embedded scientist*, which consists in translating between two language games (32), is the most widespread, the path advocated in this article appears to be more fruitful and, above all, more sustainable (33). It overcomes the negative cultural relationship that the sporting field has with the novelty and uncertainty inherent in the scientific approach, by aiming to place greater value on long-term performance rather than the quest for quick results. For practitioners, this means accepting possible discomfort, deterioration and even occasional regression—these “disturbances” (34) being necessary for a longer-term transformation of their practices. In this context, scientists do not place themselves “at the service” of sport, in a utilitarian relationship. On the contrary, they enter into a unique partnership which, over the course of the research process, transforms them as much as the practitioners with whom they work. Consubstantiality is intrinsic to their respective involvements: it is important to stress that the transformation is necessarily shared. It is different by nature, but there can be no transformation of practitioners without transformation of scientists, and vice versa.

As researchers are also engaged in a quest for short-term answers, particularly under the pressure of publication requirements, the question of the sustainability of the construction of scientific knowledge is all the more acute in the light of this type of “transformative partnership” between researchers and practitioners. In this sense, the implementation of “real-world labs”, as proposed by Wäsche et al. (35), seems to be particularly effective in encouraging the participation of various stakeholders in the processes of transformation and experimentation. It is on this condition that these partnerships can contribute fully to the sustainable development of sports and scientific practices. Finally, the limits highlighted in this way of conducting research (e.g., difficulty in generalizing, resistance from practitioners) seem to represent key challenges for the development of this type of partnership in sports science.

## Data Availability

The raw data supporting the conclusions of this article will be made available by the authors, without undue reservation.

## Footnotes

^1^This thesis, entitled “Nurturing a Sense of Community in Youth Rugby: Design and Experimentation of a Training Program for Volunteer Coaches”, is being conducted in education and training science and began in 2023. Abstract: In youth rugby, many coaches are volunteers, sometimes parents, with varying levels of knowledge, experience, and familiarity with the sport. In addition, existing coach training programs often overshadow the educational (and therefore “ethical”) aspects of the coach's mission. However, this is a crucial aspect, as training activity inevitably conveys implicit moral standards. The aim of the present research is, on the one hand, to define an educational plan based on John Dewey's democratic ethics (working with his concept of “miniature community”), and, on the other hand, through the teaching of “job rules”, to train volunteer youth coaches, making them capable of translating this ethical framework into training activity and competition. Inspired by the “rugby of movement” methodology and drawing on cultural anthropology, we implemented a transformative set-up within the under-12 category of a professional rugby club and analyzed its impact on both youth coaches and players.

^2^This research protocol was conducted as part of a doctoral thesis in education and training sciences defended in 2022 and entitled “A study of a training program for the use of digital technologies by coaches to support the learning of collective actions by players” (19). Abstract: Digital technologies appear as a way to support learning of collective actions, given their complex nature. However, the use of these technologies is not self-evident. Conceptualization allowed us to define training activities, supported by digital technologies, encouraging the learning of collective actions by the players. Four soccer coaches were consequently instructed to formalize, teach, supervise and evaluate collective actions using digital technologies. The findings highlight that these uses enhance the coaches’ activities carried out in their practice context, despite some deviations in expected activities. Moreover, coaches assess positively the success of collective actions by the players. The discussion aims to identify appropriate training conditions for the development of varied and articulated uses of digital technologies to support the learning of collective actions.
